# How to ensure equitable access to eye health for children with disabilities

**Published:** 2016

**Authors:** Hannah Kuper

**Affiliations:** Co-director: International Centre for Evidence in Disability, London School of Hygiene and Tropical Medicine, London, UK

**Figure:**
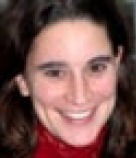
Hannah Kuper

All children need access to good quality eye care, and this must include children with disabilities.

Childhood disability is very common. The World Health Organization (WHO) estimates that there are at least 93 million children with disabilities worldwide, which equates to one in twenty children.^1^ Childhood disability is particularly common in low- and middle-income countries.

Children with disabilities may have a particularly high need for eye health services. This is because eye problems are a common cause of disability in children, and children with disabilities are particularly vulnerable to eye problems. For instance, one in three children with cerebral palsy experiences visual impairment.

Eye health services will exclude many children if they are not accessible to children with disabilities or if they are not proactive about ensuring inclusion. Eyesight is very important for all children, even more so when children have other impairments, such as those who are deaf or hard of hearing.

Even though children with disabilities have a greater need for eye health, they may not have equal access to these services, because they face a number of barriers, including:

- **Financial barriers**, e.g. paying for travel or services, since children with disabilities are more likely to come from poor households.^1^
- **Physical barriers** that limit access to buildings or transport
- **Attitudinal barriers**, e.g. when children with disabilities are seen as less worthy of attention by their own families or health workers.
- **Communication**, e.g. for children who are deaf or hard of hearing or who have intellectual impairments/learning difficulties.

Eye health services therefore need to be strengthened to ensure that children with disabilities have equal access, and this must cover all the different activities (e.g. screening, outreach, outpatient, counselling, medical and surgical treatment, and referral to other services).

The first step in ensuring that children with disabilities have equal access to eye health is to understand the different difficulties they face in accessing services in your setting. Children with disabilities are not all the same. For example, children who are deaf or hard of hearing will face different difficulties in accessing eye services compared to children with physical impairments or those with intellectual impairments. It is therefore very important to work with people with disabilities and their families in the community to find solutions together. As the disability movement says: ‘Nothing about us without us.’ In order to do this, it is useful to link with a local disabled persons organisation or other people with experience of living with disability.

This consultation process will identify specific things which can be strengthened in your setting, from which a disability plan of action can be made.^2^

Clinics must be made physically accessible for children with disabilities. The child should be able to enter the building and access the clinics, toilets and washing facilities. It is also important that equipment can be used to examine and treat children with disabilities, so that they can receive the same quality of treatment as everyone else. Ideally, physical accessibility should be considered when the clinic is being built, but there is much that can be done to improve existing facilities.

Outreach eye care services for children are often conducted in schools. However, many children with disabilities are not enrolled in school, and so will miss out. Linkages can be made with local community health workers and also with rehabilitation programmes so that children with disabilities who don't go to school can still be included.

**Figure:**
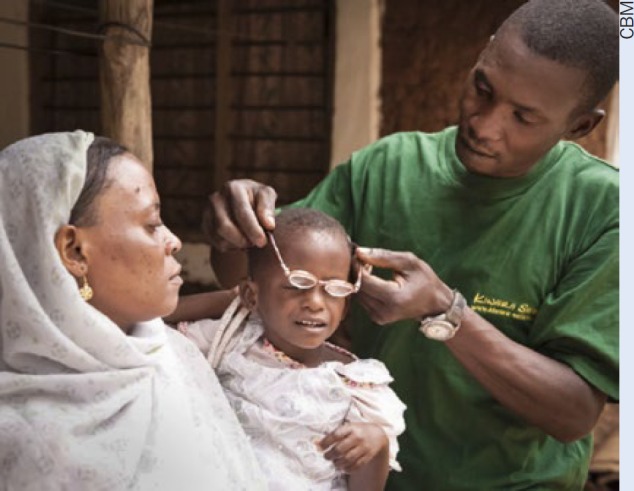

Amina is profoundly deaf and then found her eyesight was deteriorating; this made communication difficult as she had relied on her sight for lip reading. Following an eye examination, Amina had surgery for congenital cataracts and received spectacles. She is also receiving rehabilitation to assist her with both her deafness and remaining vision impairment. These intervention activities will greatly improve Amina's ongoing opportunities in social inclusion, education and the chance of decent work as an adult.

Staff must be trained in disability awareness to make sure that they interact well with children with disabilities and their families. This can include raising awareness about the rights of children with disabilities to have access to eye services, challenging negative attitudes, and offering practical training on communication with children with disabilities. Eye health workers can also receive the encouragement that the care they provide may change the whole life of those children, resulting in their inclusion in education, livelihoods and social opportunities. Local Disabled People's Organisations may be able to deliver, or participate in, the training.

Systems can be strengthened to help overcome cost barriers for children with disabilities, as for other marginalised patients. For instance, transport services may be set up or subsidised to help children with disabilities.

Formulating a plan is the first step, but it must be carried out. It can be useful to establish a disability committee or focal person to oversee the implementation of the plan and to develop a specific disability policy. It is important to designate a budget line for disability inclusion to cover the costs. The plan and policy may evolve overtime, so the programme must be reviewed regularly to make sure it is constantly being strengthened.

VISION 2020: The Right to Sight aims for all services to be equitable, and so must include children with disabilities. Furthermore, if children with disabilities do not have equal access to eye health then this violates their right to health care, and may also deprive them of life-long opportunities. It is the responsibility of all eye health workers to make sure that children with disabilities are fully included in their services.
